# Cytokine profiling in plasma distinguishes the histological inflammatory subtype of head and neck squamous cell carcinoma and a novel regulatory role of osteopontin

**DOI:** 10.3389/froh.2022.993638

**Published:** 2022-09-12

**Authors:** Ioana Ghita, Evangelia Piperi, Sergei P. Atamas, Soren M. Bentzen, Robert A. Ord, Donita Dyalram, Joshua E. Lubek, Rania H. Younis

**Affiliations:** ^1^ Department of Oncology and Diagnostic Sciences, Division of Oral and Maxillofacial Pathology, University of Maryland School of Dentistry, Baltimore, MD, United States; ^2^ Department of Oral Medicine / Pathology and Hospital Dentistry, School of Dentistry, National and Kapodistrian University of Athens, Athens, Greece; ^3^ Department of Medicine, Division of Rheumatology and Clinical Immunology, University of Maryland School of Medicine. Baltimore, MD, United States; ^4^ Department of Epidemiology and Public Health, Division of Biostatistics and Bioinformatics, University of Maryland School of Medicine. Baltimore, MD, United States; ^5^ Biostatistics Core, Institute of Clinical and Translational Research, University of Maryland, Baltimore, MD, United States; ^6^ Biostatistics Division, University of Maryland Greenebaum Comprehensive Cancer Center, Baltimore, MD, United States; ^7^ Department of Oral and Maxillofacial Surgery, University of Maryland School of Dentistry, Baltimore, MD, United States; ^8^ Head and Neck Surgery Department of Oral and Maxillofacial Surgery, University of Maryland Greenebaum Comprehensive Cancer Center, Baltimore, MD, United States; ^9^ Division of Tumor immunology and Immunotherapy, University of Maryland Greenebaum Comprehensive Cancer Center, Baltimore, MD, United States

**Keywords:** HNSCC, soluble cytokines, histologically inflamed, histologically immune excluded, Semaphorin 4D, osteopontin

## Abstract

Head and neck squamous cell carcinoma (HNSCC) can be classified according to the histological inflammatory subtype (HIS) into inflamed (HIS-INF) or immune excluded (HIS-IE). HIS-IE was previously associated with higher levels of soluble Semaphorin 4D (HsS4D) in plasma, and higher transcriptional levels of osteopontin (OPN) in the tumor tissue, compared to HIS-INF. The goal of the current study is to investigate whether the HIS inflammatory subtype can be distinguished by a differential cytokine panel in peripheral blood. Retrospectively collected five HIS-INF and five HIS-IE tumor tissue with paired plasma were included in the study. Five healthy donors (HD) and five autoimmune/chronic inflammatory conditions (AI/CI) were controls. The ELISA-Luminex™ system was used to detect 40 traditional cytokines in plasma. Human cytokine array (104 cytokines) was used for the conditioned medium (CM) of the HNSCC HN6 cell line. Semaphorin 4D (Sema4D) siRNA and recombinant human osteopontin (rh-OPN) were used to investigate the effect of OPN on Sema4D expression. The HIS-IE cytokine profile was higher than HIS-INF but comparable to AI/CI. HIS-INF had the lowest cytokine levels. HIS-IE was differentially higher in IP-10 and IL8 compared to HD, while HIS-INF was higher in IL-10. Sema4D inhibition in HN6 resulted in a decrease of OPN in the CM of HN6, and treatment with rh-OPN rescued Sema4D in HN6 cell lysate and associated CM. In conclusion, the current work demonstrates a novel association between the HIS subtypes and a differential pattern of cytokine expression in plasma. These findings can open new avenues for HNSCC patient stratification and hence provide better personalized treatment.

## Introduction

Squamous cell carcinoma (SCC) is the most common malignancy of the head and neck. Despite all advances in diagnostic and therapeutic measures, the overall 5-year survival rate stays at ∼60% and can be dismal for recurrent and advanced stages (1). Immunotherapy represents the most recent advent in the treatment of cancer. However, solid malignancies demonstrate resistance with an approximately 15% response rate in head and neck squamous cell carcinoma (HNSCC) (2–4). Hence, further understanding of HNSCC inflammatory phenotypes is warranted.

Different strategies describing HNSCC tumor inflammation have been reported in the literature. These ranged from biomarkers expression *in situ* in the tumor tissue to soluble inflammatory cytokines in body fluids (5–9). Cytokines like interferon gamma-induced protein 10 (IP-10, or CXCL10) were shown to be higher in plasma of early oral SCC (OSCC) compared to healthy controls (8), while interleukin 6 (IL-6), IL8, and vascular endothelial growth factor (VEGF) as well as IL-1β and tumor necrosis factor alpha (TNF*α*) were detected in the serum of HNSCC patients (9). IL-6 seemed to be most sensitive to early-stage OSCC patients (10). IL-1alpha, IL-6, IL-8, VEGF, granulocyte-macrophage colony-stimulating factor (GM-CSF), and basic fibroblast growth factor (FGF) were detected in the supernatants of SCC cell lines, and supernatants of freshly isolated primary HNSCC cultures (9).

HNSCC can be stratified according to the histological inflammatory subtype (HIS), i.e., inflamed (HIS-INF) and immune excluded (HIS-IE) (6). Recent reports showed a significant correlation between the HIS-IE tumor tissue and higher levels of the soluble immune biomarker Semaphorin 4D (HsS4D) in plasma, compared to HIS-INF (6, 11). Semaphorin 4D (Sema4D) (a.k.a. CD100 or immune Semaphorin) is a transmembrane glycoprotein belonging to the fourth group of the Semaphorins family. It can function as a cell-bound protein or in a cleaved soluble form (sSema4D) (6, 12). Sema4D is expressed by almost all inflammatory cells (13–15) and plays a role in the pathogenesis of several autoimmune conditions, allergies, and chronic inflammation (16–18). Sema4D has been linked to poor prognosis in sarcomas, non-small-cell lung cancer, ovarian cancer, and colorectal cancer (19–22). It can be expressed in the tumor cells and the infiltrating immune cells (23). HNSCC with Sema4D-positive tumor cells correlated with dense noninflamed peritumoral stroma (6), and *in vitro* models showed that HNSCC produced sSema4D can upregulate myeloid-derived suppressor cells (MDSC) (24–26) and plays a role in extracellular matrix (ECM) deposition by fibroblasts (11).

Osteopontin (OPN) [a.k.a. secreted phosphoprotein (SPP1)] is a secreted, multifunctional, ECM, calcium-binding, glycosylated phosphoprotein implicated in several normal biological functions and pathologic processes (27). OPN is produced physiologically by a broad range of cells and has been identified in various body fluids, playing a key role in bone remodeling and vascularization. OPN is also expressed by several immune cells, acting as a chemotactic factor, as a mediator of cell activation and cytokine production, and an antiapoptotic factor, regulating inflammation and immune responses (28). OPN can be involved in the tumorigenesis of several cancer types, including HNSCC acting through multiple signaling pathways to promote cell proliferation, adhesion, invasion, and migration (29, 30). Interestingly, higher transcriptional levels of OPN were detected in HNSCC tumor tissue that is associated with HsS4D in plasma (6).

Here, we present a differential pattern of cytokine expression in plasma associated with the HIS subtypes of HNSCC and describe a novel regulatory role of OPN in regulating Sema4D, which is implicated in the pathogenesis of the HIS-IE phenotype. These findings can open new avenues for HNSCC stratification to enhance personalized care and improve patient outcomes.

## Materials and methods

### Tumor tissue and plasma samples

Plasma and tumor tissue pairs of 10 HNSCC patients retrospectively collected upon obtaining informed consent according to the Institutional Review Board (IRB) (HP-00073603) of the University of Maryland School of Medicine (UMSOM) were used in the study (6). The samples were selected to include five HIS-IE and five HIS-INF tumors. The formalin-fixed paraffin-embedded tumor tissue was processed by the University of Maryland, Baltimore (UMB), Pathology Biorepository Services. Blood was collected at the time of patient presentation for surgery and plasma was prepared within 2 h of collection. Five autoimmune/chronic inflammatory (AI/CI) conditions and five healthy donors (HD) served as controls according to the UMSOM IRB protocol (HP-00074877) or were purchased from Innovative Research (Novi, MI, United States).

Sema4D immunohistochemical (IHC) staining was described before (6), using Sema4D primary antibody (clone 30/CD100) (BD Transduction Laboratories) and secondary antibody (BA-9200), followed by the Vectastain Elite ABC kit (PK-6102, mouse IgG) (Vector Laboratories, CA, United States). Scoring for the HIS phenotype was carried out using the Aperio ImageScope software. sSema4D levels were previously detected using the direct ELISA technique (6), human CD100 primary antibody (1:100) (clone: 133-1C6; Invitrogen, eBioscience), Goat anti-mouse IgM-Heavy chain, HRP conjugate (Invitrogen USA, IL, United States; cat. # 62-6820), and recombinant human (rh) CD100 (catalog #310-29) (PeproTech) for standards (detection limit 3.1–1000 ng/ml, >155 ng/ml cut-off for HsS4D).

### Luminex bead-based multiplex assay

The Luminex bead-based multiplex assay was performed at the University of Maryland Center for Innovative Biomedical Resources, Cytokine Laboratory. The Human Cytokine/Chemokine Luminex™ System (Panel A38—Millipore Sigma) Millipore kit #HCYTMAG-60K was used to detect the 38 T-helper 1 (Th1) and Th2 cytokines/chemokines in plasma following the manufacturer recommendations: EGF, FGF-2, EOTAXIN, TGF-A, GCSF, FLT-3L, GM-CSF, FRATALKINE, IFNA2, IFN-G, GRO, IL-10, MCP-3, IL-12 P40, MDC, IL-12 P70, IL-13, IL-15, CD40L, IL-17A, IL-1RA, IL-1A, IL-9, IL-1B, IL-2, IL-3, IL-4, IL-5, IL-6, IL-7, IL-8, IP-10, MCP-1, MIP-1A, MIP-1B, TNF-A, TNF-B, VEGF, and two of Th17 cytokines/chemokines: IL-17 E and IL-17F. TGFB-MAG-64K was used for the detection of TGF-B1 levels. The samples were run as duplicates, for each patient or control. All plates contained high and low control. The plates were read using a Luminex MagPix reader.

### Tissue culture and reagents

HNSCC cell lines WSU-HN6 (T3N2bM0) and WSU-HN4 (T4N1M0) of the base of the tongue, NOKSI (normal oral-keratinocytes), and DOK (potentially malignant cell lines) were used. WSU-HN6 and NOKSI were DNA authenticated at the Johns Hopkins Genetic Resources Core Facility, Baltimore. DOK was a gift from Dr. Abraham Schneider. Cells were cultured in Dulbecco's modified Eagle medium containing fetal bovine serum. Conditioned medium (CM) was concentrated using Millipore Amicon Ultra centrifugal filter units. rh-OPN protein (cat. # 120-35) was obtained from PeproTech and serially diluted in the medium for treatment. The siRNA system Hs_SEMA4D_6 (catalog no. SI03053701) (20 nM) and HiPerFect transfection reagent (catalog no.301704) (Qiagen Inc., Germantown, MD, United States) were used for Sema4D gene silencing.

### Western blot

The CM were concentrated and harvested, as well as the cells, in SDS cell lysis buffer with the protease inhibitor tablet (catalog no. 11836170001) (Roche Diagnostics, Indianapolis, IN, United States). The whole-cell lysate was separated using SDS-PAGE Western Blot. Primary antibodies used were Sema4D (30/CD100, catalog no. 610670; BD Biosciences/Pharmingen), osteopontin (Cat # 889-656-7625, Rockland), and GAPDH (8C2, sc-81545; Santa Cruz Biotechnology). The secondary antibodies were anti-rabbit IgG (catalog no. sc-2301) and anti-mouse IgG (catalog no. sc-2302) (Santa Cruz Biotechnology). Image J software was used for the immunoblot quantification analyses.

### *In vitro* cytokine analyses

Human Cytokine Array Kit from R/D System (cat # ARY022B) for 104 cytokines’ detection in CM was used according to the manufacturer protocol. Concentrated CMs were added in duplicates to the activated membranes overnight. Membranes were washed and the detection antibody cocktail was added and incubated for 1 h at room temperature. Streptavidin–HRP was added to each membrane and incubated for 30 min at room temperature on a shaker. Chemi Reagent Mix was used for exposure.

### Statistical analysis

For the distribution of the 40 plasma cytokines among the groups, the two-group comparison was carried out using the *t*-Test or Mann–Whitney *U* test. For multiple comparisons using independent samples, the Kruskal–Wallis test was used. Statistical significance was considered a *P*-value of less than 0.05 (*), less than 0.01 was (**) or <0.0001 (***). Prism/GraphPad software was used for statistical analyses. No mathematical correction was made for multiple comparisons. The global cytokine cluster gram was plotted using MORPHEUS Versatile matrix visualization and analysis software (https://software.broadinstitute.org/morpheus).

## Results

### HNSCC and the histological pattern of inflammation

**Figure 1:**
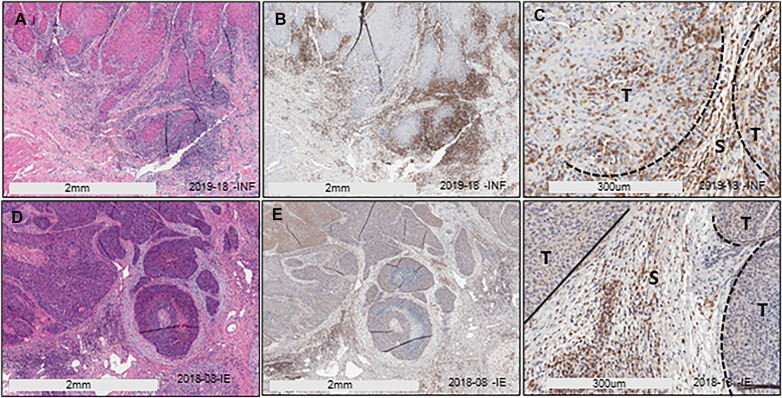
HIS stratification in HNSCC. (**A**) H/E stain of HIS-INF OSCC. (**B**,**C**) IHC of Sema4D illustrating HIS-INF. (**D**) H/E stain of HIS-IE SCC on the base of the tongue. (**E**,**F**) IHC of Sema4D illustrating HIS-IE (**E**: low power; **F**: higher power). HIS, histological inflammatory subtype, HIS-INF, HIS inflamed, HIS-IE, HIS immune excluded, T, tumor islands, S, stroma.

We have previously described two main HIS subtypes in HNSCC depending on whether the tumor-associated inflammatory cells infiltrate into the tumor islands (HIS-INF) or are excluded (HIS-IE) (6). Sema4D immune biomarker stain highlighted the inflammatory cell infiltrate. Here, we wanted to investigate if these two HIS subtypes would have differential levels of inflammatory cytokines expression in the peripheral blood. To answer this question, we used 10 cases of previously collected HNSCC tumor tissue and its paired plasma (6) (Table 1). Plasma from HD and AI/CI conditions were used as controls. One case of HIS-INF was excluded because, in addition to HNSCC, the patient was diagnosed with gout. HIS-INF demonstrated the immune cells infiltrating into the core of the tumor islands (Figures 1A–C). HIS-IE showed very few to no immune cells infiltrating into the tumor islands, with some cases showing peritumoral stromal fibrotic or fibromyxoid rim (Figures 1D–F). The sSema4D level in peripheral blood was predetermined (6), with the HIS-IE cases expressing HsS4D in plasma (>155 ng/ml) and the HIS-INF expressing lower levels of sSema4D (LsS4D) in plasma (≤155 ng/ml).

**Table 1:** Demographics and clinical information of the HNSCC samples, HD, and AI/CI controls.

HNSCC	HIS-IE, *N* (%)	HIS-INF, *N* (%)	*P*-value
Age			0.480
Median (range)	67 (52–80)	62 (51–69)	
Gender			0.999
M	3 (60%)	3 (60%)	
F	2 (40%)	2 (40%)	
Race			0.999
Caucasian	4 (80%)	4 (80%)	
African American	1 (20%)	1 (20%)	
Stage			0.485
I	3 (60%)	5 (100%)	
II	1 (20%)	0 (0%)	
III	0 (0%)	0 (0%)	
IV	1 (20%)	0 (0%)	
Location			0.999
Oral and mobile tongue	3 (60%)	5 (100%)	
Oropharyngeal	2 (40%)	0 (0%)	
HPV			0.999
Positive	1 (20%)	0 (0%)	
Negative	4 (80%)	5 (100%)	
Smoking			0.444
Yes	5 (100%)	3 (60%)	
No	0 (0%)	2 (40%)	
Alcohol			0.99
Yes	2 (40%)	1 (20%)	
No	3 (60%)	4 (80%)	
Recurrence			0.99
Yes	0 (0%)	1 (20%)	
No	5 (100%)	4 (80%)	
sSema4D			0.007
LsS4D	0 (0%)	5 (100%)	
HsS4D	5 (100%)	0 (0%)	
sSema4D, ng/ml			0.005
Median (range)	453 (282–710)	50 (36–70)	

Controls	HD, *N* (%)	AI/ CI, *N* (%)	*P*-value
Age	** **	** **	0.126
Median (range)	31 (21–59)	55 (38–72)	
Gender			0.999
M	2 (40%)	3 (60%)	
F	3 (60%)	2 (40%)	
Race			0.999
Caucasian	3 (60%)	2 (40%)	
African American	1 (20%)	3 (60%)	
Hispanic	1 (20%)	0 (0%)	
sSema4D			0.0079
LsS4D	5 (100%)	0 (0%)	
HsS4D	0 (0%)	5 (100%)	
sSema4D, ng/ml			0.038
Median (range)	68 (32–90)	195 (114–323)	

Fisher’s exact test was used for categorical variables. Two-way ANOVA, for age, stage, and sSema4D. Five cases of AI/CI: myasthenia gravis, sarcoidosis, asthma, rheumatoid arthritis with acute tonsilitis, and rheumatoid arthritis.

HNSCC, head and neck squamous cell carcinoma; HIS-IE, histological inflammatory subtype immune excluded; HIS-INF, histological inflammatory subtype inflamed; HD, healthy donors, AI/CI, autoimmune/chronic inflammation.

### Traditional cytokine panel in plasma of HIS-INF and HIS-IE HNSCC

To investigate the differential level of expression of soluble inflammatory cytokines in plasma of HIS-INF and HIS-IE, we used the ELISA-Luminex™ system. Macrophage-derived chemokine (MDC) was excluded from the analysis because its values were beyond the detection limit except for the HIS-INF group. IL-17 F and IL3 were also excluded because the values were lower than the detection limit of the assay in the HIS-INF.

**Figure 2:**
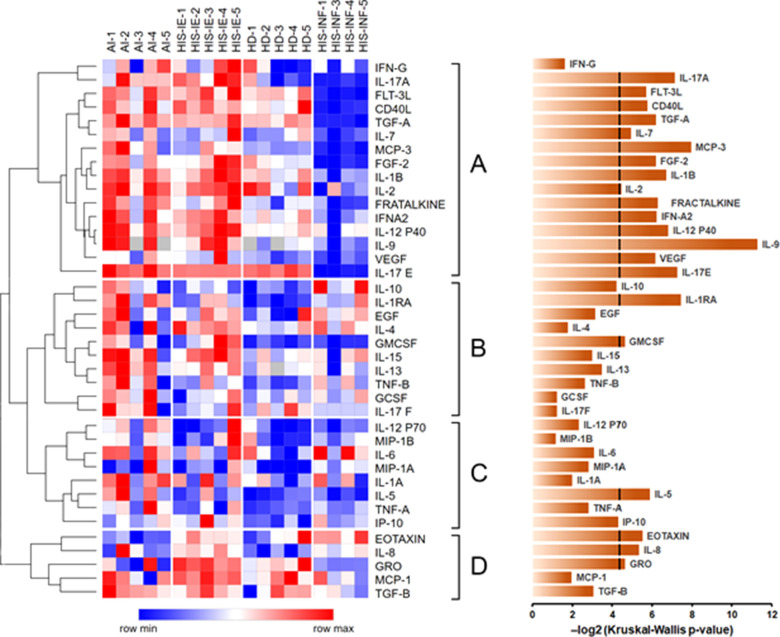
Global cytokine differential expression in plasma among HD, AI/CI, HIS-IE, HD, and HIS-IFN. Heatmap of log_2_-transformed cytokine concentrations measured in the tested samples. Cytokines were clustered using Spearman rank correlation with complete linkage. The major clusters are sequentially labeled A–D to the right of the heatmap. The heatmap was constructed using Morpheus. The bar graph on the right shows negative log_2_-transformed *p*-values for each cytokine based on the Kruskal–Wallis analysis across the four groups. The line crossing some of the bars at the 4.322 value corresponds to the *p*-value of 0.05. HD, healthy donors, AI, autoimmune, HIS-INF, HIS inflamed, HIS-IE, HIS immune excluded.

The values of each cytokine were normalized to the corresponding values of HD for global observation. The distribution of most of the cytokines was highest in the AI/CI group followed by HIS-IE and then HD. Most of the cytokines were lowest in the HIS-INF group except for Eotaxin, IL6, and IL-10 (Figure 2, Supplementary Figure S1, and Supplementary Table). There were significant differences between the groups for all cytokines in cluster A (Figure 2) except for IFN-γ. In other clusters, GM-CSF, IL-5, Eotaxin, IL-8, and GRO were also different between the groups, whereas string tendency to significance was observed for IL-10 (*p* = 0.0538) and IP-10 (*p* = 0.0507) (Figure 2).

**Figure 3:**
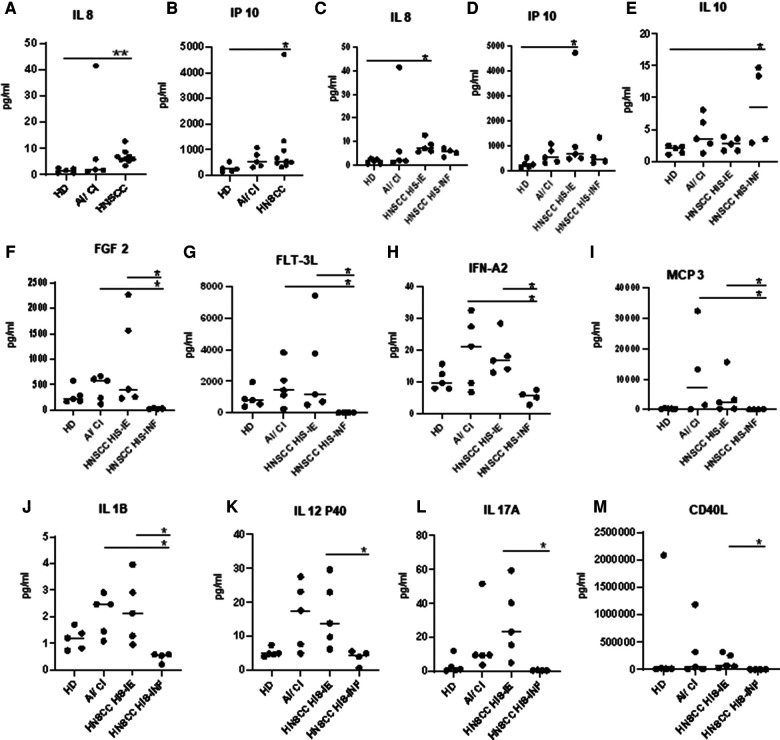
Plasma cytokine levels of HNSCC versus HD. (**A**,**B)** Plasma cytokines demonstrating a significant difference in expression between HNSCC and HD. (**C–E**) or upon HIS stratification and HD. (**F–M**) Differential cytokine expression between HIS-IE and HIS-INF. Multiple comparison analysis using the Kruskal–Wallis test.

Multiple comparison analyses of HD, AI/CI, and HNSCC showed that the HNSCC group was higher than the HD in IL-8, IP10 (CXCL10), and IL-1RA (Figures 3A,B). Under two-group analyses, only IL-8 maintained significance (Supplementary Table). Upon HNSCC HIS stratification, IL-8 and IP-10 were revealed to be higher in HIS-IE (*p* = 0.028 and 0.0496, respectively), and IL-10 to be higher in HIS-INF compared to HD (*p* = 0.048) (Figures 3C–E). IL-10 maintained significance in the two-group comparison (Supplementary Table).

HIS-IE was similar to the AI/CI and significantly higher than HIS-INF in most of the cytokines (cluster A) using multiple comparisons and/or two-group analyses. The same cytokine cluster was lowest in HIS-INF. Also, GRO, MCP-1 (Cluster D), and IL2 showed higher levels in HIS-IE compared to HIS-INF (Figures 2, 3F–M, and Supplementary Table).

The AI/CI group was significantly higher in IL-1RA, IL-1A, and IL1B in addition to Fractalkine, IL12P40, and IL9 from cluster A, compared to the HD, using two-group analyses (Supplementary Table). IL-1RA, IL-5, IL-9, and IL-17E were higher in AI/CI compared to HNSCC and most retained significance using multiple comparisons (Supplementary Figures S2A–C). Upon HNSCC HIS stratification, most of the aforementioned cytokines were significantly higher in AI/CI compared to HIS-INF but not HIS-IE. Only Eotaxin was significantly lower in the AI/CI group compared to the HNSCC even after HIS stratification (Supplementary Figures S2D–L and Supplementary Table).

### Sema4D plays a role in HNSCC HN6 inflammatory cytokine profile

Sema4D-positive tumor cells were associated with noninflamed dense stroma (6, 11). *In vitro*, studies have shown that the HN6 cell line associated with Sema4D plays a role in the upregulation of MDSC (24, 26) and that Sema4D inhibition in HN6 causes downregulation of extracellular matrix deposition by fibroblasts (11), suggesting HN6 cell line as an *in vitro* model mimicking the Sema4D-positive HIS-IE tumor cells. This triggered us to investigate if Sema4D plays a role in the inflammatory cytokine profile associated with HN6. To answer this question, we first tested the soluble cytokines in the CM of NOKSI compared to the HN6 cell line. HN6 showed higher levels of GRO*α*, osteopontin (OPN, SPP1), FGF19, M-CSF, pentraxin 3 (PTX-3), Vit D BP, and macrophage inhibitory cytokine 1 (MIC-1) compared to NOKSI. IL-1α, IL1-RA, angiogenin, angiopoietin 2, soluble interleukin 1 receptor-like 1 (ST2), transferrin receptor protein 1 (TFR), PDGF-AB/BB, and PDGF-AA were higher in the CM of NOKSI compared to the HN6 (Supplementary Figure S3).

**Figure 4:**
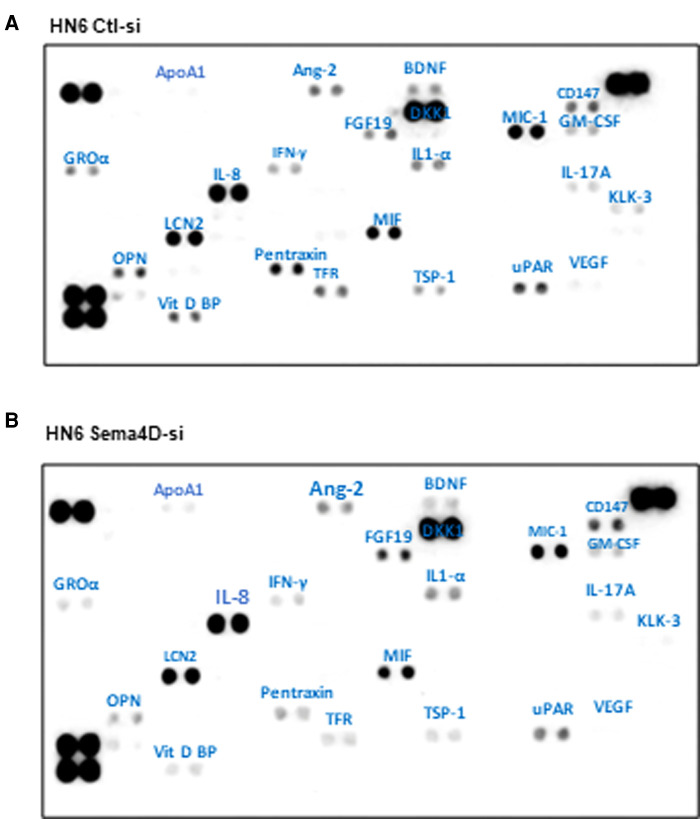
*In vitro* cytokine expression in HNSCC HN6 cell line upon Sema4D inhibition. (**A**) CM of HNSCC HN6 cell line after 3 days of control SiRNA transfection versus, (**B**) Sema4D siRNA transfection. CM, conditioned medium.

To explore the effect of Sema4D on the inflammatory cytokine production by HNSCC, we transfected the HN6 cell line with Sema4D-siRNA and collected the CM after three days. Sema4D inhibition resulted in a significant downregulation of several cytokines produced in the CM of HN6 including GRO-α, OPN, Vit D BP, PTX-3, and brain-derived neurotrophic factor (BDNF), in addition to a slight reduction in TFR1, thrombospondin-1 (THBS1, TSP-1), kallikrein 3 (PSA, KLK3), and urokinase-type plasminogen activator receptor (uPAR) (Figure 4).

### Role of OPN in the regulation of Sema4D in HNSCC HN6 cell line

**Figure 5:**
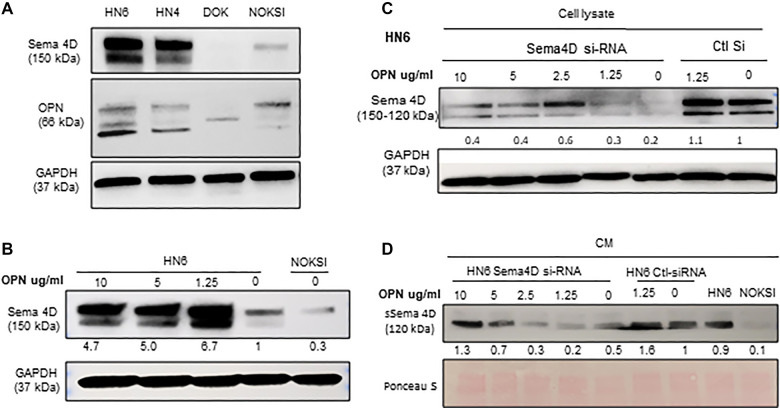
OPN plays a role in Sema4D regulation in the HNSCC HN6 cell line. (**A**) Immunoblot analysis demonstrates the basal level of OPN in HN6, HN4, DOK, and NOKSI. (**B**) Treatment of HNSCC with hr-OPN at different doses upregulates Sema4D in the cell lysate. (**C**) Sema4D inhibition in HN6 using Sema4D-siRNA 20 nM. OPN rescues Sema4D expression in HN6 cell lysates and (**D**) in the CM, after Sema4D-siRNA.

We have previously described that HsS4D correlated with HIS-IE and higher transcriptional level of OPN in HNSCC tumor tissue, compared with tumors with LsS4D (6). To investigate if OPN plays a role in the regulation of Sema4D, we checked the basal level of OPN in HN6 and HN4 cancer cell lines, NOKSI, and potentially malignant cell lines DOK. Interestingly, the immunoblot showed that Sema4D and OPN were overexpressed in HN4 and HN6 compared to NOKSI and DOK (Figure 5A). Then, we tested if treatment of the HN6 cell line with rh-OPN protein would affect Sema4D expression. Interestingly, Sema4D significantly increased in the tumor cell upon treatment with OPN starting at a low concentration (Figure 5B). To further investigate if OPN would rescue Sema4D expression upon inhibition, we transfected the HN6 cell line with Sema4D-siRNA. Indeed, OPN treatment rescued Sema4D expression in the Sema4D-siRNA HN6 cell lysate, and increased sSema4D produced in the CM was noticed, especially at higher concentrations of OPN treatment that corresponded with less intracellular repertoire (Figures 5C,D).

## Discussion

**Figure 6:**
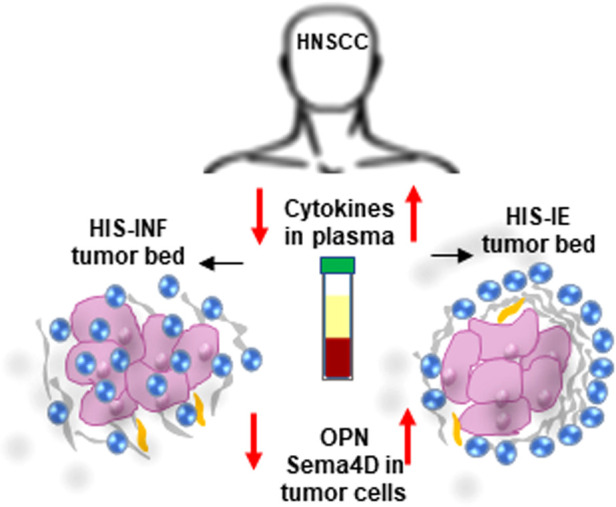
Differential cytokine expression in plasma associated with HIS subtypes of HNSCC. Low cytokine levels in plasma associate with HIS-INF while elevated cytokine levels associate with HIS-IE subtype. Sema4D and OPN-positive feedback are higher in HIS-IE.

The current work illustrates that HNSCC HIS subtypes, can present with a differential cytokine profile in blood, reflecting a distinct underlying disease mechanism (Figure 6). Interestingly, the HIS-INF was the lowest in plasma cytokines compared to all groups. This suggests that in the HIS-INF subtype, the immune system recruits the inflammatory cells to the tumor bed, to an extent that it drains the peripheral circulation rendering the patient in a systemic immune suppressed status. On the other hand, the higher proinflammatory cytokines in plasma of the HIS-IE suggest that the excluded inflammatory cells at the tumor bed keep sending positive chemotactic signals that can be detected in the peripheral circulation. Whether these two HIS subtypes are independent or a continuum is yet to be investigated.

In considering the soluble cytokine expression in peripheral blood of HNSCC, attention to underlying AI/CI would be warranted (18). Comparing the chronic inflammation and fibrotic nature of most of the collagenous autoimmune diseases (31) to the peritumoral fibrotic rim and excluding inflammatory cells in the HIS-IE, as well as the shared cytokine cluster observed between the AI/CI and HIS-IE, suggest that HIS-IE may adopt a chronic inflammatory process similar in part to autoimmunity. More patients bearing HNSCC and AI/CI diagnosis need to be investigated if both cytokine profiles can be discerned in the same patient. IL-1RA, a physiological inhibitor of preformed IL-1, can have a significant translational aspect in this context, since our data showed it is highest in AI/CI followed by HNSCC compared to HD, indicating underlying chronic pathological condition. Interestingly, blockade of IL-1RA demonstrated a central role in controlling several autoimmune diseases and protects from Th2 skewing of the immune responses (32–34).

Our data are in concordance with previous reports detecting higher levels of IL10, IP-10 (CXCL10), and IL-8 in peripheral blood of HNSCC compared to HD (7–9). Yet, the HIS stratification model illustrated in the current work revealed differential expression of IL10 in the HIS-INF, and IP-10, and IL8 in HIS-IE compared to HD. IL-10 is a key anti-inflammatory immune suppressive cytokine that inhibits myeloid cells and IFN-α activation (35), while IP-10 is a cytokine secreted in response to IFN-γ by several cells, which may include monocytes, endothelial cells, and fibroblasts. IP-10 has a proinflammatory role as a chemoattractant to T cells, NK cells, monocytes/macrophages, and dendritic cells. IP-10 is involved in several autoimmune conditions (36, 37) and chronic infectious diseases (38). In lung cancer, the degree of malignancy has been correlated with the level of secretion of IP-10 by the tumor. Less progressive lung carcinoma secretes more IP-10 (39). Whether this is true in HNSCC needs to be investigated further. It is worth mentioning that previous transcriptional analysis demonstrated that the HIS-IE tumor bed expresses a lower IFN-γ gene and associated signature, compared to HIS-INF (6). IL-8 is a proangiogenic factor and is a chemoattractant to neutrophils (40). Moreover, other cytokines like GRO and MCP-1, grouped with IL-8 in cluster D of the heat map of the current work, were higher in HIS-IE and are predominantly myeloid chemoattractants. GRO has a neutrophil chemoattractant activity and it decreased significantly upon inhibition of Sema4D in the HN6 cell line. These findings are in concordance with previous transcriptional analysis that showed myeloid cells to be higher in the tumor bed of HIS-IE/HsS4D, especially neutrophils compared to HIS-INF/LsS4D (6).

In addition, previous transcriptional analysis demonstrated a higher hypoxic phenotype of the HIS-IE/HsS4D (6). Hypoxia induces several inflammatory and proangiogenic cytokines. Cluster A included several proangiogenic cytokines (41). Among them, IL1b is a major mediator of innate immunity and plays a central role in several human autoinflammatory conditions and malignancies. It has been reported to be more expressed in invasive carcinomas and correlated with lung cancer incidence (42). In addition, the VEGF and matrix metalloproteinases (MMPs) were shown to act synergistically with OPN to induce a metastatic phenotype on some cancer cells and promote angiogenesis and invasion (43, 44). Additional angiogenic and hematopoietic growth factors of cluster A cytokines are FMS-like tyrosine kinase 3 ligand (FLT-3L) and CD40L (45). FLT-3L is a hematopoietic growth factor; its receptor is upregulated under hypoxia and can propagate B cell growth and dysfunction. Vaccinations against FLT-3L and GM-CSF can protect from malignancies formation in murine models (46). CD40L also propagates B cell development and germinal centers, is constitutively expressed in B cells and myeloid cells, and is finally regarded as a potential immune checkpoint therapeutic target (47).

Other cytokines differentially expressed in cluster A can further explain the histological features of HIS-IE. FGF2 is interestingly associated with resistance to antiangiogenic therapy, poor survival (48), and increased tumor fibrosis, which can impede intratumoral drug accumulation (49). In addition, IL12p40 induces a negative feedback loop by competitively binding to the IL-12 receptor and is also a chemoattractant for macrophages (50), which can be induced upon activation of NK cells and macrophages against HNSCC (51). IL9 is mainly involved in autoimmunity, allergic reactions, and parasitic infections, and is a growth factor of T cells and mast cells; it has received increasing attention to the role of IL-9-skewed CD8+ T (Tc9) cells, mast cells, and Vδ2 T cell-derived IL-9 in tumor immunity (52, 53). IFN-α is a major player in innate immune responses. Recent reports suggest immunostimulating IFN-α as an attractive target to restore the TH2 HNSCC immune microenvironment (35). The HNSCC suppression of IFN-α, although mainly due to several cytokines acting synergistically, the HNSCC micro milieu was shown to severely depress IFN-α secretion, specifically through IL-10 alone but not IL-8 (35). That is in concordance with the differential expression of IFN-α, and IL-8 in the HIS-IE group, vs. IL-10 in HIS-INF, which is observed in the current study.

The current *in vitro* data demonstrate a novel role of OPN in the regulation of Sema4D expression in HNSCC as well as suggest a positive feedback loop between the two molecules. We previously demonstrated an almost ten-fold increase in the OPN (SPP1) transcriptional level in tumor tissue of HsS4D phenotype compared to LsS4D and about a fourfold increase in MMP7, which is implicated in OPN cleavage (6). That HIS-IE tumor tissue correlated with HsS4D in plasma and positive Sema4D tumor cells. We also described the HN6 cell line associated with Sema4D as an *in vitro* model for HNSCC upregulation of MDSCs and extracellular stromal density (11, 24). Taken together, these findings suggest that OPN and Sema4D play a role in modulating the HIS-IE phenotype.

Specifically, in OSCC, elevated levels of OPN at the tumor-free surgical margins have proven to be predictive of recurrence (43). OPN is well known to facilitate wound healing/fibrosis through regulation, and differentiation of fibroblasts and myofibroblasts (54). Overexpression of OPN by both tumor and stromal cells has been correlated with reduced survival and drug resistance to cisplatin and 5-fluorouracil (55). Further investigation of the role of OPN and Sema4D in HNSCC drug resistance would be warranted.

There is increasing evidence that restoration of the HNSCC-induced immune bias could be improved by the inhibition of immune cell cytokine receptors (35). The current work presents a pilot model for detecting a soluble cytokine panel in plasma that is differentially associated with HIS subtypes in the tumor bed. This may bear translational potential that can open new avenues for patient stratification and personalized treatment in the field of HNSCC.

## Data Availability

The original contributions presented in the study are included in the article/**Supplementary Material**, further inquiries can be directed to the corresponding author.
