# Supplementary figures and images for: Cytokine profiling in plasma distinguishes the histological inflammatory subtype of head and neck squamous cell carcinoma and a novel regulatory role of osteopontin
Source: Front Oral Health. 2022 Sep 12;3:993638. doi: 10.3389/froh.2022.993638 (PMC9632968; doi:10.3389/froh.2022.993638)

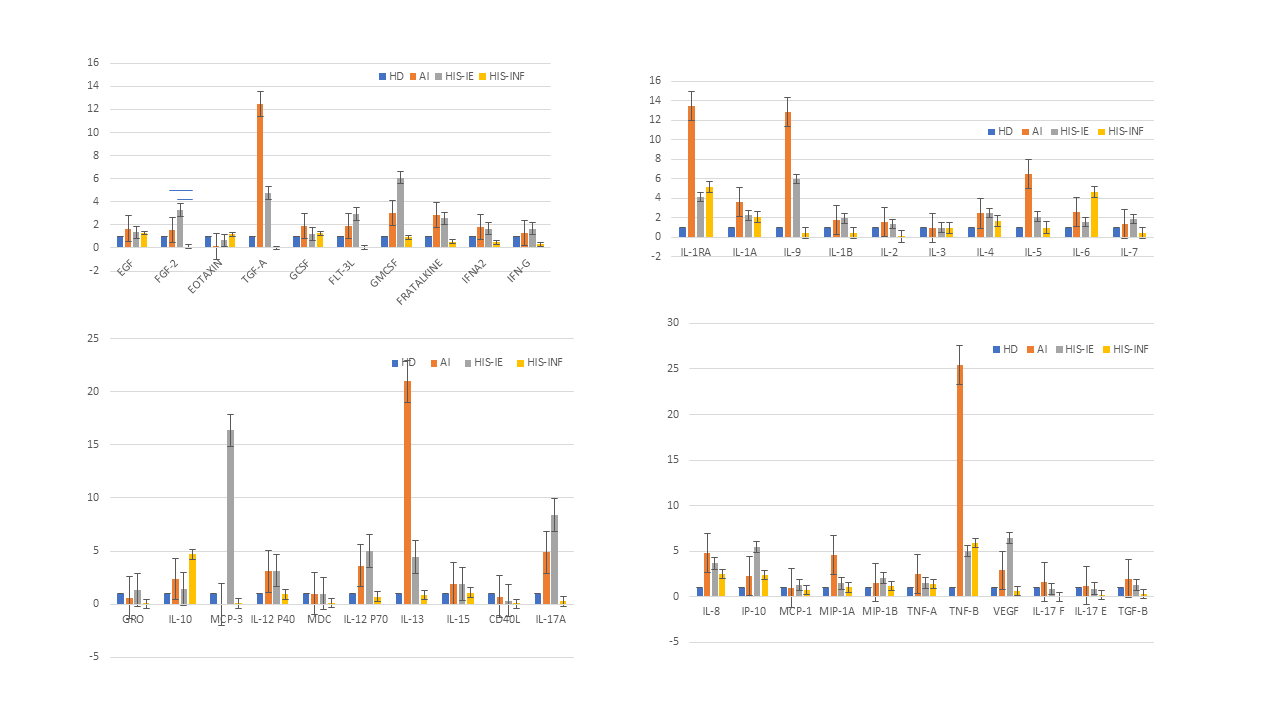

Supplement: Supplementary file 2 [file Image1.tif]

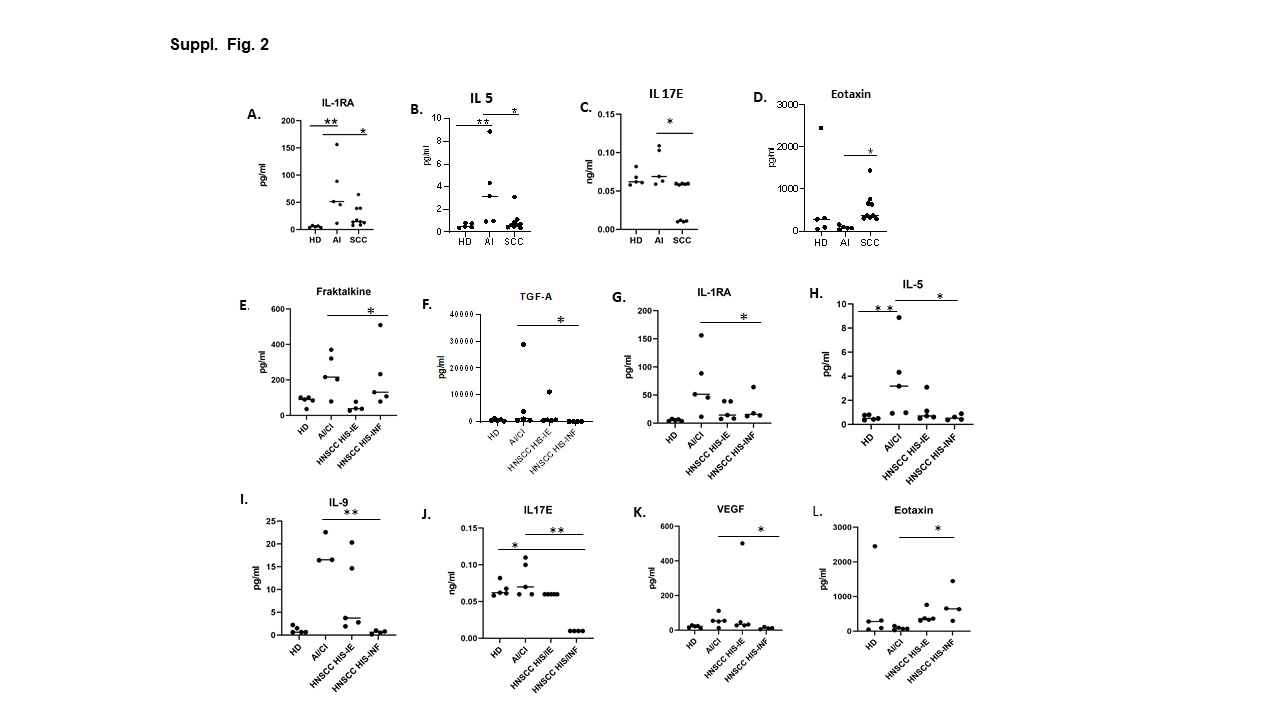

Supplement: Supplementary file 3 [file Image2.tif]

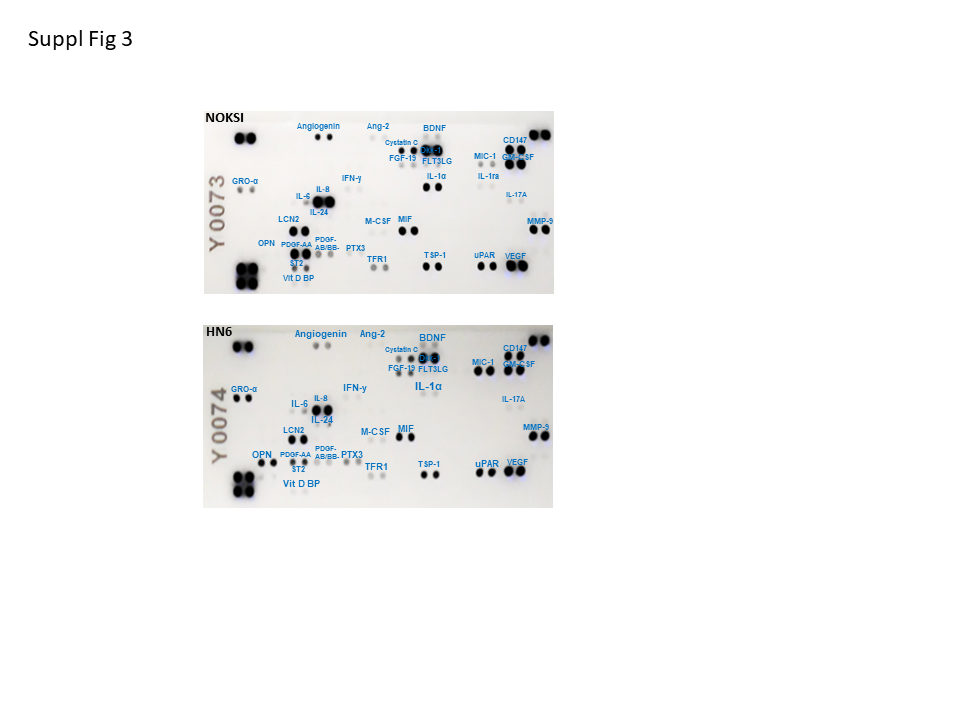

Supplement: Supplementary file 4 [file Image3.tif]
